# An overall and dose-response meta-analysis for thyrotropin and thyroid cancer risk by histological type

**DOI:** 10.18632/oncotarget.10282

**Published:** 2016-06-24

**Authors:** Na Hu, Zhan-Ming Li, Jin-Feng Liu, Zhen-Zhen Zhang, Li-Shun Wang

**Affiliations:** ^1^ Institute of Biomedical Sciences, Minhang Hospital, Fudan University, Shanghai, P.R. China; ^2^ School of Public Health Taishan Medical University, Shandong, P.R. China; ^3^ Ruijin Hospital, Shanghai Jiao-Tong University School of Medicine, Shanghai, P.R. China

**Keywords:** thyroid cancer, serum thyrotropin, meta-analysis, differentiated thyroid carcinoma, papillary thyroid carcinoma

## Abstract

Thyrotropin (TSH) is thought as a risk factor for thyroid cancer. However, the effect of serum TSH might depend on histological types of thyroid cancer. We searched for related studies including serum TSH as an exposure and thyroid cancer as a result in PUBMED, EMBASE and Chinese National Knowledge Infrastructure up to April 21, 2016. This meta-analysis included 22 articles with 53,538 participants. When comparing all histological thyroid cancer, the pooled odds ratios of thyroid cancer in patients with nodules was found to increase significantly with higher serum TSH concentrations for differentiated thyroid carcinoma (1.88 *vs* .1.48, *P* = 0.0000) and papillary thyroid carcinoma (2.08 *vs.* 1.48, *P* = 0.0006). Each 1 mU/L increase of serum TSH was associated with 14% greater risk of thyroid cancer for all histological thyroid cancer, 16% for differentiated thyroid carcinoma and 22% for papillary thyroid carcinoma. In addition, high serum TSH was associated with a reduced risk for follicular thyroid carcinoma (OR = 0.73, 95% CI: 0.52, 1.02). This meta-analysis suggested high serum TSH concentration is risky for papillary thyroid carcinoma but not for follicular thyroid carcinoma.

## INTRODUCTION

Thyroid cancer is the most common malignant tumor of the endocrine system [1]. In 2003, the American Cancer Society reported an incidence of 1/10000 in the USA. Notably, the incidence of thyroid cancer is rising faster than any other malignancy [1]. Thyroid carcinoma according to the histological type can be classified as differentiated and undifferentiated. The most frequent types include papillary thyroid carcinoma (PTC), follicular thyroid carcinoma (FTC), differentiated thyroid carcinoma (DTC) [2].

Thyrotropin (TSH) is the major growth factor for thyroid cells. TSH plays a role in thyroid growth and organogenesis [3, 4]. It has been reported that higher serum TSH concentration is associated with an increased risk of thyroid cancer [5]. TSH is thought to play roles in carcinogenesis [6, 7] and suppression therapy of TSH using thyroid hormone is widely used for DTC [8, 9]. However, the risk of thyroid malignancy to serum TSH might depend on histological types. In order to address this issue, the patients with thyroid nodule and different histological thyroid cancer, including DTC, PTC as well as FTC, were included to analyze the association of thyroid cancer and serum TSH. An overall and dose-response meta-analysis were performed for this serum TSH and thyroid cancer risk by histological type.

## RESULTS

### Literature search and study characteristics

**Figure 1:**
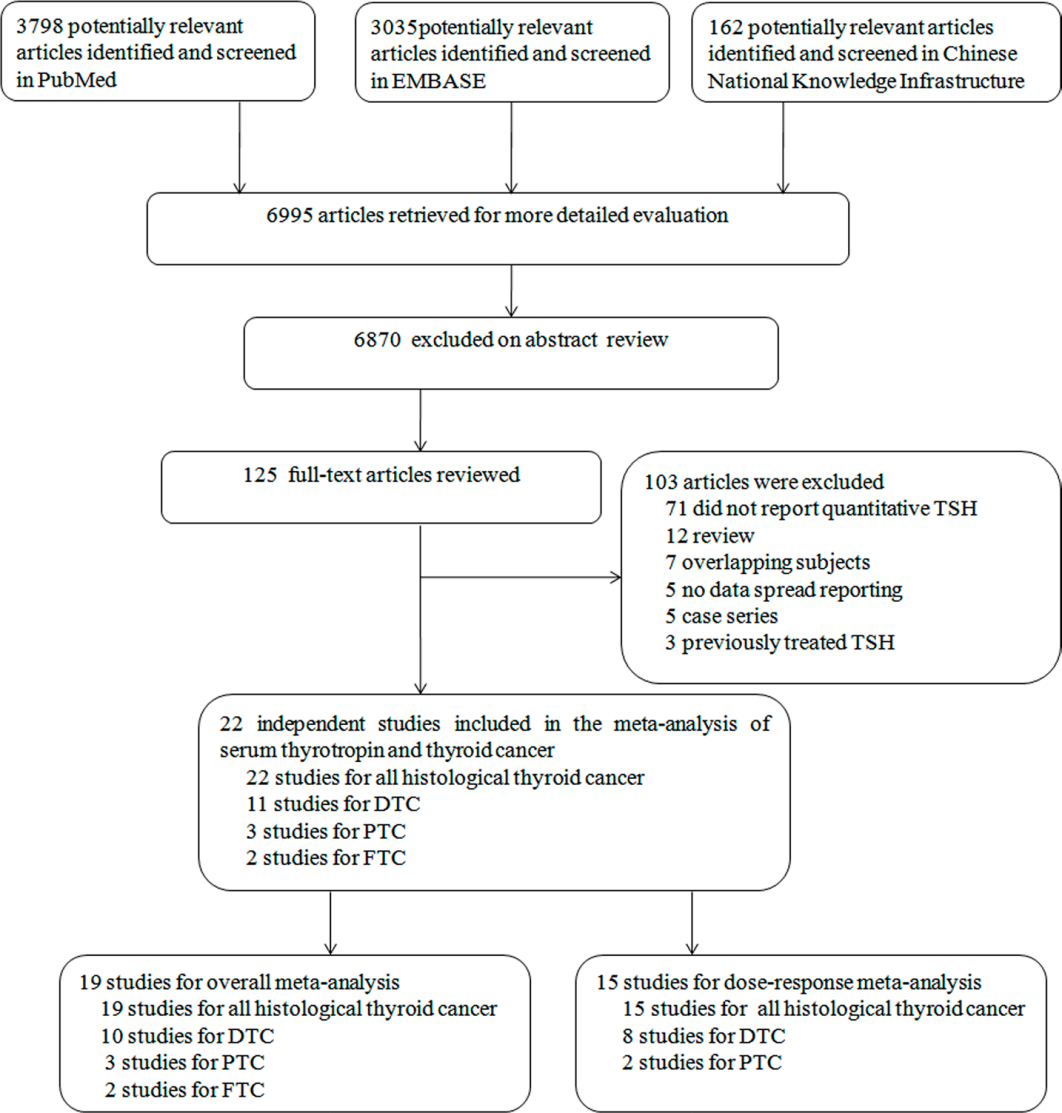
Flowchart of publication selection for the meta-analysis

Of 6995 articles obtained on a literature search, 125 papers passed the screening phase with the full-text articles reviewed (Figure 1). This yielded a final total of 22 studies [1, 7, 10–29] being combined in meta-analysis (53,538 participants). The most common reasons for study rejection included: studies did not report quantitative TSH (*n* = 71), the articles were reviews (*n* = 12) or overlapping subjects (*n* = 7). The duration of follow-up ranged from 1 to 20 years. Among these studies, 22 studies [1, 7, 10–29] for overall thyroid cancer, 10 studies [1, 8, 15, 17, 19, 21, 22, 24, 28, 29] for differentiated thyroid carcinoma, 3 studies [1, 15, 28] for papillary thyroid carcinoma, and 2 studies [19, 24] for follicular thyroid carcinoma. Main characteristics of the studies are shown in Table 1. Newcastle-Ottawa Scale (NOS) was analyzed for quality assessment and all these included studies were above 6 stars (Supplementary Table S1).

**Table 1:** Overview of included studies

First author (country, year)	Study type	Mean age	Percent female subject	Numbers of cases/total analyzed	Duration of follow-up	Control group	Types of thyroid cancer included (%)	TSH category (mU/liter)	OR for TSH category (95% CI)	Covariates adjusted for in analysis	Overall meta-analysis included	Dose-response analysis included	Quality score
Boelaert et al. (UK, 2006) [10]	Prosp. cross-sect	47.8	86.9	92/1183	18	Thyroid nodule patients	All histological thyroid cancer	< 0.4 0.4–0.9 1.0–1.7 1.8–5.5 > 5.5	1 1.31 (0.45–3.81) 2.72 (1.02–7.27) 3.88 (1.48–10.19) 11.18 (3.23–38.63)	Age, goiter type, and serum TSH concentration	Y	Y	9
Haymart et al. (UK, 2008) [11]	Retrosp. cross-sect	46	80.8	212/735	13	Benign surgical patients	PTC (87%) FTC/HCC (7%) Other(6%)	< 0.06 0.06–0.39 0.40–1.39 1.40–2.49 2.50–4.99 > 5.00	1 1.65 (0.59–4.60) 1.39 (0.59–3.27) 2.50 (1.04–6.04) 3.52 (1.37–9.02) 4.56 (1.35–15.45)	Age, nodule size, and preoperative serum	Y	Y	9
Jonklaas et al. (USA, 2008) [12]	Retrosp. cross-sect	49	74	17/50	3.5	Benign surgical patients	PTC(88%) FTC (12%)	0.34–1.1 1.2–2.1 2.1–2.8 > 2.8	1 8.6(2.0–35.9) 6.0 (0.6–55.7) (No cases)	None	N	Y	7
Polyzos et al. (Greece, 2008) [13]	Retrosp. cross-sect	48.2	86	36/383	16	Thyroid nodule patients	PTC (69%) FTC/HCC(17%) Other (14%)	< 0.4 0.4–0.8 0.9–1.4 1.5– 4.0 > 4.0	1 0.60 (0.18–1.97) 0.98 (0.33–2.91) 2.54 (0.99–6.52) 0.63 (0.07–5.49)	None (excluded multivariate analysis)	Y	Y	8
Fiore et al. (Italy, 2009) [1]	Prosp. cross-sect	49.2	80.3	504/10178	7	benign thyroid nodular disease	PTC (100%)	< 0.4 0.4–0.59 0.6–0.89 0.9–1.3 1.31–3.4 > 3.4	1 0.80 (0.51–1.27) 2.01 (1.46–2.77) 2.66 (1.98–3.58) 4.29 (3.17–5.08) 3.50 (2.10–5.83)	None (excluded patients taking levothyroxine)	Y	Y	7
Fiore et al. (Italy, 2010) [15]	Prosp. cross-sect	40	81.2	1275/27914	12	Thyroid nodule patients	PTC (100%)	< 0.4 0.4–0.59 0.6–0.89 0.9–1.3 1.31–3.4 > 3.4	1 1.79 (1.42–2.23) 2.72 (2.24–3.3) 3.76 (3.12–4.53) 5.32 (4.45–.36) 10.36 (6.34–16.89)	None	Y	Y	8
Gul et al. (Turkey, 2010) [17]	Retrosp. cross-sect	45.5	78.7	166/441	3	Benign surgical patients	PTC (89%) FTC/HCC(11%)	0.4–0.62 0.63–1.67 1.68–4.0	1 2.37 (1.34–4.19) 5.74 (3.03–10.89)	Age, gender, nodule type	Y	Y	8
Dorange et al. (France, 2011) [22]	Retrosp. cross-sect	44	80.9	47/94	20	Benign surgical disease	PTC (79%) FTC/HCC (21%)	0.1–1.0 1.01–2.0 2.01–4.5	1 3.43 (1.37–8.57) 11.67 (2.21–61.48)	Matched on age, gender, ethnicity, method of TSH measurement	N	Y	9
Rio et al. (Brazil, 2011) [23]	Retrosp. cross-sect	49.9	89	62/144	2	Benign surgical patients	PTC (87%) FTC/HCC (7%) Other (6%)	< 0.4 0.4–1.39 1.4–2.49 2.5–4.49 4.5–5.5	1 1.33 (0.31–5.81) 1.67(0.37–7.53) 2.2(0.43–11.22) 1.33(0.14–12.82)	None	Y	Y	7
Ding et al (China, 2011) [21]	Retrosp. cross-sect	48.3	None	218/956	8	Benign surgical patients	All histological thyroid cancer	<0.27 0.27–1.39 1.40–2.29 2.23–4.20 > 4.20	1 0.81(0.39-1.66) 1.01(0.49-2.06) 1.81(0.88-3.71) 2.03(0.82-5.03)	None	Y	Y	7
Zafon et al. (Spain, 2012) [26]	Retrosp. cross-sect	53.8	80.9	76/386	3	Benign surgical patients	PTC (96%) FTC (4%)	< 0.4 0.4–4 > 4	1 1.91(0.93– 3.92) 5.38(1.75–16.58)	None	Y	Y	6
Kim et al. (Korea, 2010) [19]	Retrosp. cross-sect	50.9	75.8	296/1638	2	benign thyroid nodular disease	PTC (99%) Other (1%)	< 0.17 0.17–1.17 1.18–2.01 2.02–4.05 > 4.06	1 0.99 (0.27–3.63) 1.44 (0.93–2.23) 1.72 (1.12–2.63) 1.98 (1.06–3.70)	Age, gender, nodule size, nodule type, thyroid autoimmunity	Y	Y	8
Haymart et al. (USA, 2009) [14]	Retrosp. cross-sect	48.9	80.0	212/735	13	Benign surgical patients	PTC (52%) FTC/HCC (37%) Other (11%)	< 0.06 0.06–0.39 0.4–1.39 1.4–2.49 2.5–4.99 > = 5.0	1 1.65 (0.59–4.6) 1.39 (0.59–3.27) 2.5 (1.04–6.04) 3.52 (1.37–9.02) 4.56 (1.35–15.45)	Gender, age, nodule size, and preoperative serum TSH concentration	N	Y	8
Kim et al. (Korea, 2013) [27]	Prosp. cross-sect	47.1	81.7	2184/3905	7	healthy controls	PTC (96.6%) FTC (1.4%) Other (2%)	0.40 ≤ 1.10 1.11 ≤ 1.63 1.64 ≤ 2.30 2.31 ≤ 4.80	1 1.27 (1.03–1.57) 1.55 (1.25–1.92) 2.21 (1.78–2.74)	Age, sex, and the presence of a family history of thyroid cancer	N	Y	9
Jin et al. (USA, 2010) [18]	Retrosp. cross-sect	49	86	135/660	18	Thyroid Nodule patients	PTC (87%) FTC (9%) Other (4%)	<0.9 0.9-1.7 1.8-5.5 > 5.5	1 2.05 (1.25–3.35) 2.36 (1.31–4.25) 6.17 (1.77–21.46)	Age and sex	Y	Y	7
Kim et al. (Korea, 2012) [7]	Retrosp. cross-sect	48.2	80.1	52/1329	4	Benign surgical patients	PTC (98%) FTC (2%)	Continuous	0.70 (0.47–1.03)	None	Y	N	8
Lee et al.(Korea, 2012) [24]	Retrosp. cross-sect	None	None	35/164	7	Benign surgical patients	PTC (54%) FTC (46%)	Continuous	0.804 (0.410–1.575)	None	Y	N	7
Jiao et al. (China, 2015) [28]	Retrosp. cross-sect	49.2	75.6	113/365	2	Benign surgical patients	PTC (100%)	Continuous	1.52 (1.01–2.42)	None	Y	N	7
Nixon et al. (USA, 2010) [20]	Retrosp. cross-sect.	55	75.0	111/156	1	Thyroid Nodule patients	All histological thyroid cancer	Continuous	3.53 (1.35–9.24)	None	Y	N	8
Moon et al. (Korea, 2012) [25]	Retrosp. cross-sect	53.5	84.1	42/483	1	Thyroid Nodule patients	All histological thyroid cancer	Continuous	1.402 (1.018–1.932)	None(exclude multivariate analysis)	Y	N	9
Maia et al. (Brazil, 2011) [7]	Retrosp. Cross-sect.	47.2	84.6	50/143	10	Benign surgical patients	All histological thyroid cancer	Continuous	1.03 (0.97–1.08)	Age, gender, nodule type	Y	N	8
Gerschpacher et al. (Austria, 2010) [16]	Retrosp. cross-sect	55	44.8	33/87	14	Medullary cancer, C cell hyperplasia	All histological thyroid cancer	Continuous	0.86 (0.58–1.25)	Age, gender	Y	N	8

Prosp.cross-sect, prospective cross-sectional; Retrosp. cross-sect, retrospective cross-sectional; OR, odds ratio; N/A, not available; CI, confidence interval; Y, included; N, not included.

### Quantitative synthesis

#### Overall meta-analyses

**Figure 2:**
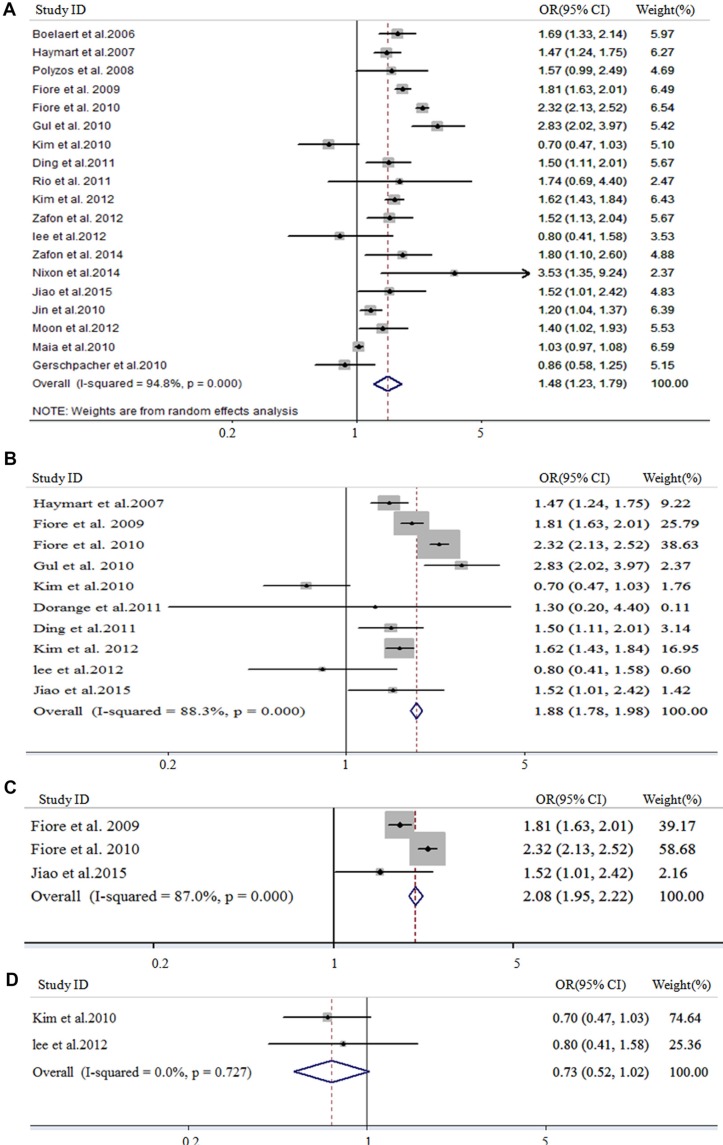
Forest plot for study-specific and pooled OR in overall meta-analysis The size of each grey square is proportional to the study's weight calculated as inverse of variance. OR, odd ratio; 95% CI, 95% confidence intervals; thyroid cancer, all the histological types of thyroid cancer (19 studies); DTC, differentiated thyroid carcinoma (10 studies); PTC, papillary thyroid carcinoma (3 studies); FTC, follicular thyroid carcinoma (2 studies). Weights are from random effects analysis.

In meta-analysis for all histological thyroid cancer risk and serum TSH, 47,297 patients from 19 studies were involved. As shown in Figure 2A, the pooled OR for all histological thyroid cancer was 1.48 (95% CI 1.23–1.79, I^2^ = 94.8%) based on the random-effects models. In meta-analysis of DTC prevalence and serum TSH, 10 studies including 43,922 patients were involved. As shown in Figure 2B, the pooled OR was 1.88 (95% CI 1.78–1.98, I^2^ = 88.3%). In meta-analysis of PTC prevalence and serum TSH, 3 studies including 38,457 patients were involved. As shown in Figure 2C, the pooled OR was 2.08 (95% CI 1.95–2.22, I^2^ = 87%). In meta-analysis of FTC prevalence and serum TSH, two studies including 1802 patients were involved. As shown in Figure 2D, high serum TSH demonstrated a reduced risk on FTC. The pooled OR for FTC patients was 0.73 (95% CI 0.52–1.02, I^2^ = 0).

**Figure 3:**
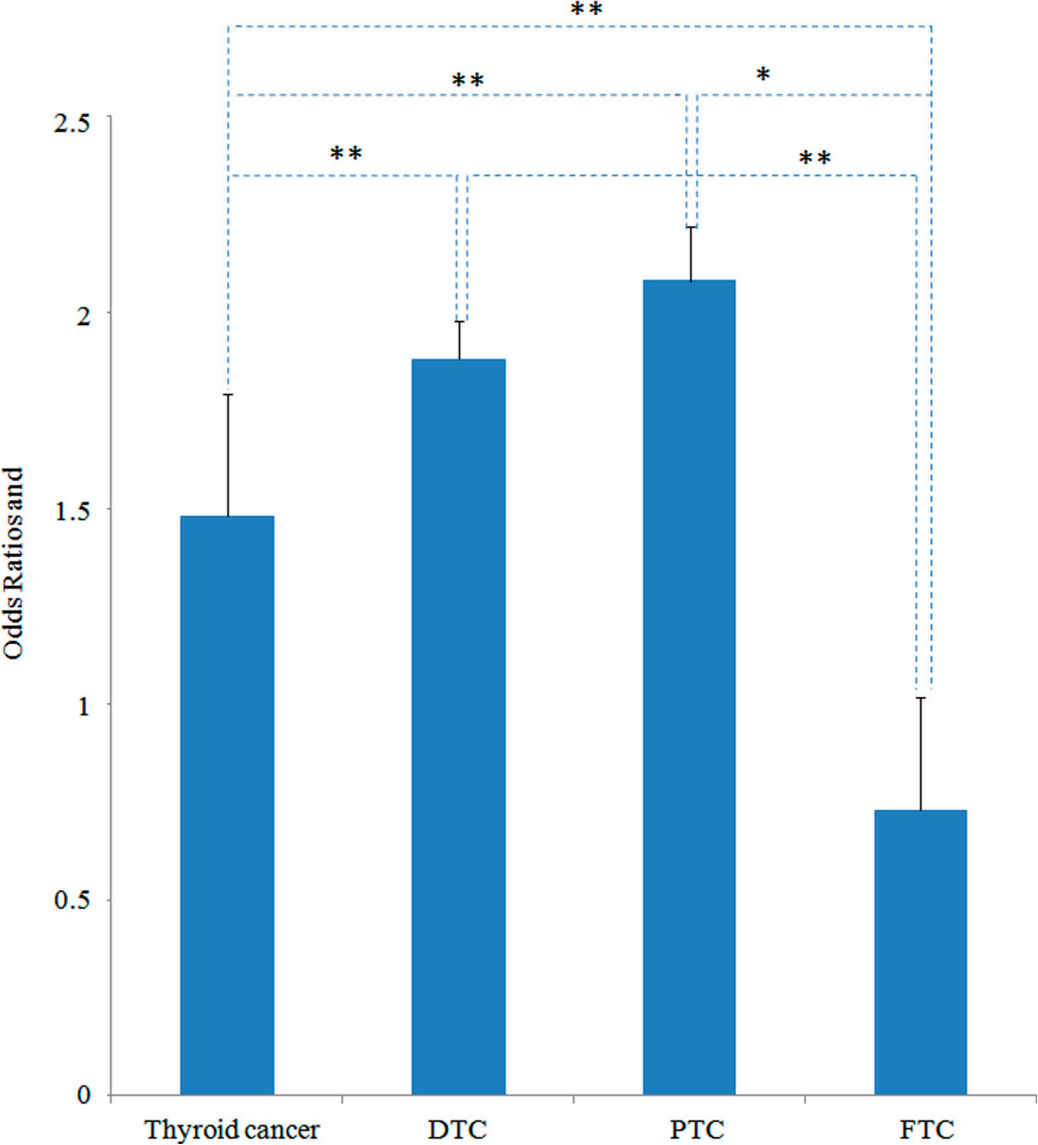
OR comparison between all histological thyroid cancer (thyroid cancer), differentiated thyroid carcinoma (DTC), papillary thyroid carcinoma (PTC) and follicular thyroid carcinoma (FTC) *P < 0.05, **P < 0.01.

When comparing all histological thyroid cancer, the pooled odds ratios of thyroid cancer in patients with nodules was found to increase significantly with higher serum TSH concentrations for differentiated thyroid carcinoma (1.88 *vs*.1.48, *P* = 0.0000) and papillary thyroid carcinoma (2.08 *vs.* 1.48, *P* = 0.0006) (Figure 3). On the contrary, the OR of FTC was significantly lower than all histological thyroid cancer (0.73 *vs.* 1.48, *P* = 0.0000), DTC (0.73 *vs*. 1.88, *P* = 0.0000) as well as PTC (0.73 *vs*. 2.08, *P* = 0.0399) (Figure 3).

### Dose-response meta-analysis

**Figure 4:**
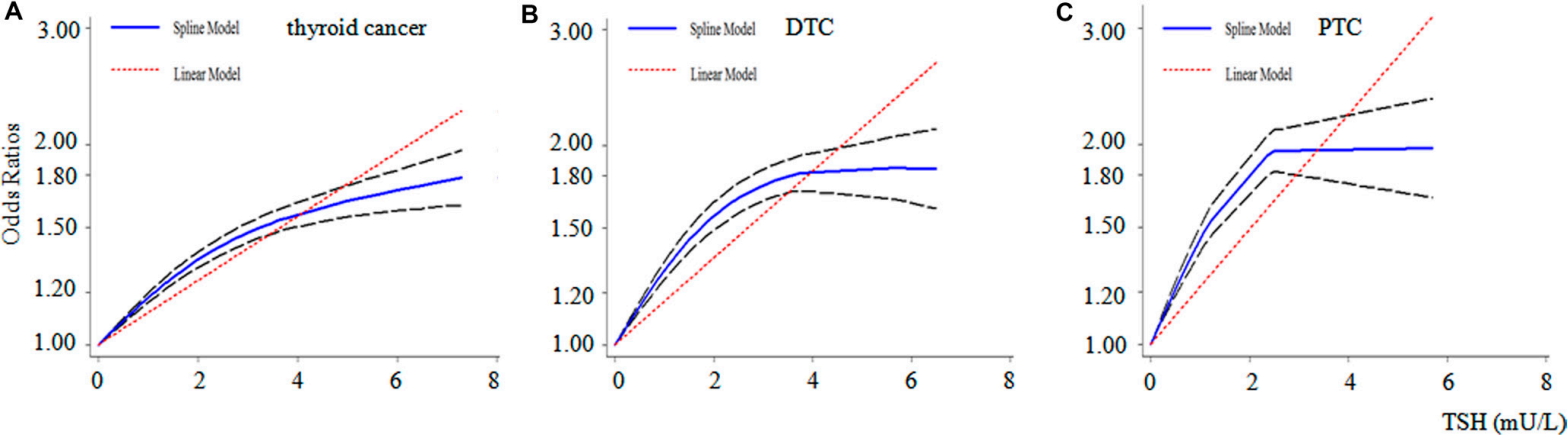
Dose-response relationship for serum TSH and thyroid cancer Dotted lines represent the 95% confidence intervals for the fitted trend. The dose-response relationship plot between TSH levels (mU/L) and different histological types of thyroid cancer. Thyroid cancer, all thyroid cancer (15 studies); DTC, differentiated thyroid carcinoma (8 studies); PTC, papillary thyroid carcinoma (2 studies).

15 studies involving 50,811 participants were included in the dose-response meta-analysis of all histological thyroid cancer risk and serum TSH. And a nonlinear relationship was found (*p* = 0.000) as shown in Figure 4A. Compared with benign thyroid disease patients, the fractional polynomial model estimates of the OR were 1.4 and 1.6 for 2.0 and 4.0 mU/L of serum TSH respectively. This meta-analysis showed a 14% increase of thyroid cancer risk for each 1 mU/L increase in serum TSH.

8 studies involving 43,675 participants were included in the dose-response meta-analysis of serum TSH and DTC. And a nonlinear relationship was found (*p* = 0.000) as shown in Figure 4B. Compared with benign thyroid disease patients, the fractional polynomial model estimates of the OR were 1.7 and 1.8 for 2.0 and 4.0 mU/L of serum TSH respectively. This meta-analysis showed a 16% increase of DTC risk for each 1 mU/L increase in serum TSH.

2 studies involving 38,092 participants were included in the dose-response meta-analysis of the serum TSH and PTC. And a nonlinear relationship was found (*p* = 0.000) as shown in Figure 4C. Compared with benign thyroid disease patients, the fractional polynomial model estimates of the OR were 1.9 and 2.0 for 2.0 and 4.0 mU/L of serum TSH respectively. This meta-analysis showed a 22% increase of PTC risk for each 1 mU/L increase in serum TSH.

### Meta-regression analyses

We conducted the meta-regression analysis for the evaluation the heterogeneity to investigate whether the association between TSH and thyroid carcinoma was modified by patient source, publication year and study type. We found that the study type can explain 45.08% heterogeneity. The overall OR of thyroid cancer calculated from prospective studies was 1.95 (95% CI 1.59–2.39). However, the corresponding OR from retrospective studies was 1.39 (95% CI 1.18–1.63), which was significantly lower than the former one (*P* < 0.001).

### Sensitivity analysis

In a sensitivity analysis in which one study at a time was removed and the rest analyzed, the pooled OR ranged from 1.93 to 2.08 for overall thyroid carcinoma, which indicated that the pooled estimates were stable and not influenced by a single study (Supplementary Table S2).

### Publication bias

No evidence for publication bias was indicated by Egger's regression test (*P* = 0.448). The funnel plot also indicated no evidence of publication bias.

## DISCUSSION

This study provided a new view for a potential relation between serum TSH and risk of thyroid cancer by overall meta-analysis and dose-response meta-analysis. This study indicated that the risk of thyroid cancer for higher serum TSH depends on histological types. Higher serum TSH was revealed to be associated with increased risk for PTC but reduced risk for FTC. Given the large number and the high prevalence of thyroid cancer worldwide [1, 12], the results from this meta-analysis have important implications. However, our result for serum TSH and FTC was based on two pre-operation studies. It needs more studies for verification.

In addition, FTC belongs to DTC and TSH suppression was widely used after DTC thyroidectomy [8, 9, 14]. Even that, this meta-analysis suggested the higher serum TSH would lead to reduced risk for FTC. TSH effect on FTC with TSH suppression treatment after operation might be different and random clinical trials for the TSH treatment on FTC would present direct evidence. In addition, further biological experiments are needed to verify this difference between PTC and FTC and reveal the molecular mechanisms under the difference. For example, the expression of TSH receptor in most DTC thyroid cancers is similar to or slightly lower than that found in normal thyrocytes. And TSH was thought promote cancer cell proliferation via its receptors [11]. However, it has been reported that the proliferation of a FTC cell line without TSH receptor expression was inhibited after ectopic expression of TSH receptor, which indicated the effect of TSH on DTC may be complicated and depends on the cell context [30].

Our meta-analysis had several strengths, including the associations for differentiated thyroid carcinoma, papillary thyroid carcinoma and follicular thyroid cancer were evaluated by categories of thyroid cancer separately. In addition, two different methods of overall and dose-response meta-analysis were used to investigate the association between thyroid cancer risk and serum TSH, which presented both the pooled and dynamic view of their relationship.

Our meta-analysis also had some limitations. A statistically significant heterogeneity between the studies was observed, which was likely to be attributed to the variation in study design. The relatively small number of studies limited our ability to identify other histological groups. Unpublished data, non-English-language studies, and missed studies may exist and may have influenced our results. Furthermore, more studies are needed on cancer stage, grade, metastasis and morbidity rate to quantify with greater confidence the nature of the relationship between TSH and risk of subgroup PTC.

In conclusion, findings from this meta-analysis of studies suggested that the risk of thyroid cancer for higher serum TSH depends on histological types. Higher serum TSH concentration is associated with increased risk of PTC but reduced risk of FTC.

## MATERIALS AND METHODS

### Search strategy

We conducted a systematic literature search using the PUBMED, EMBASE and Chinese National Knowledge Infrastructure databases to April 21, 2016. The search strategy in these databases all comprised MeSH terms of “thyroid neoplasm” or “thyroid cancer,” as well as (all fields) “thyrotropic hormone,” “thyrotropin,” “TSH,” and “hormothyrin,” following the MOOSE (Meta-analyses Of Observational Studies) guidelines [20]. No language restriction was applied. We also hand searched reference lists from eligible papers as well as reference lists from national and international thyroid cancer and nodule guidelines [31–35].

### Inclusion criteria

The criteria for inclusion were as follows: (i) observational epidemiological studies (case–control, case–cohort, or cohort) on total preoperative serum TSH levels and thyroid cancer incidence in population; (ii) the exposure was serum TSH concentration; (iii) reporting the odds ratio (OR) or relative risk (RR) estimates with the corresponding 95% confidence intervals (CI) or sufficient information to calculate them for each 1 mU/L increase in serum TSH; (IV) for those reports that included the same subjects (or overlapping subjects) as another study, the most informative study was chosen for the primary analysis. We excluded review articles, case reports and case series containing only thyroid cancer patients. Two investigators (NH and ZML) independently identified the eligible studies. Initially the article titles and abstracts were screened for potentially relevant papers, followed by the full-text review of the remaining studies (Figure 1). Discrepancies were resolved through consensus.

### Data extraction

NH, ZML, JFL independently extracted the study data and any disagreements were resolved by consensus. Reported effect measures and confidence intervals within TSH exposure categories were extracted. Where several effect measures were reported in an article, the most completely adjusted models accounting for possible confounding was chosen. The following information was also extracted from each of the eligible publications: first author's name, publication year, study location, follow-up years, age, TSH exposure categories, ascertainment of benign thyroid disease, procedures, percentage of females.

### Study quality score

Newcastle-Ottawa Scale (NOS) was analyzed for quality assessment [36] (Supplementary Table S1). The NOS awards a maximum of nine points to each case-control study: four for the quality of selection (adequate case definition, representativeness of cases, selection of controls, definition of controls), two for comparability (confounding) and three for the quality of the exposure (ascertainment of exposure, same method of ascertainment of cases and controls). It awards a maximum of nine points to each cohort study: four for the quality of selection (representativeness, selection of non-exposed cohort, ascertainment of exposure, no disease at start of study), two for comparability (confounding) and three for the quality of the outcome (assessment of outcome, length of follow-up and adequacy of follow-up). Studies with NOS values of six or greater were considered moderate to high-quality studies and those with a NOS value of less than six were regarded low-quality studies.

### Statistical analysis

Statistical analysis was performed with STATA 12.0 (Stata-Corp, College Station, TX), using two-sided hypothesis testing and alpha = 0.05. We assessed heterogeneity for both within- and between studies with the I^2^ statistic [37] as a measure of the proportion of total variation in estimates that is due to heterogeneity, where I^2^ values of 25%, 50%, and 75% correspond to cut-off points for low, moderate, and high degrees of heterogeneity [37]. We calculated pooled ORs using the random-effects models of DerSimonian and Laird for the high degree of heterogeneity [38]. Dose-response meta-analyses were conducted by using the GLST command with the generalized least-squares method for trend estimation of summarized dose-response data, based on the Greenland and Longnecker method [39]. Restricted cubic splines were used to assess for potential curvilinear relations.

Using meta-regression analysis, we further investigated whether the association between TSH and thyroid cancer risk by histological type was modified by study-specific factors, including patient source, publication year and study type. We conducted a sensitivity analysis, in which one study at a time was removed and the rest analyzed to assess whether the results were markedly affected by a single study with STATA 12.0. Evidence of publication bias was assessed by visual inspection of funnel plots using Egger's regression test [40].

## SUPPLEMENTARY MATERIALS


