# Supplementary material for: An overall and dose-response meta-analysis for thyrotropin and thyroid cancer risk by histological type
Source: Oncotarget. 2016 Jun 24;7(30):47750–9. doi: 10.18632/oncotarget.10282 (PMC5216976; doi:10.18632/oncotarget.10282)
Supplement: Supplementary file 1 [file oncotarget-07-47750-s001.pdf]

An overall and dose-response meta-analysis for thyrotropin and thyroid cancer risk by histological type

Supplementary Materials

Supplementary Table S1: Assessment of atudy quality

Studies (all cohort studies)	Quality Indicators From Newcastle-Ottawa Scale								
	1	2	3	4	5A	5B	6	7	8
Boelaert et al. (UK) [10]	☆	☆	☆	☆	☆	☆	☆	☆	☆
Haymart et al. (UK) [11]	☆	☆	☆	☆	☆	☆	☆	☆	☆
Jonklaas et al. (USA) [12]	☆		☆	☆	☆	☆	☆		☆
Polyzos et al. (Greece) [13]	☆		☆	☆	☆	☆	☆	☆	☆
Fiore et al. (Italy) [1]			☆	☆	☆	☆	☆	☆	☆
Fiore et al. (Italy) [15]		☆	☆	☆	☆	☆	☆	☆	☆
Gul et al. (Turkey) [17]	☆	☆	☆	☆	☆	☆	☆		☆
Dorange et al. (France) [22]	☆	☆	☆	☆	☆	☆	☆	☆	☆
Rio et al. (Brazil) [23]	☆	☆	☆	☆		☆	☆		☆
Ding et al (China) [21]	☆	☆	☆	☆			☆	☆	☆
Zafon et al. (Spain) [26]	☆	☆	☆	☆			☆		☆
Kim et al. (Korea) [19]	☆	☆	☆	☆	☆	☆	☆		☆
Haymart et al.(USA) [14]	☆		☆	☆	☆	☆	☆	☆	☆
Kim et al. (Korea) [27]	☆	☆	☆	☆	☆	☆	☆	☆	☆
Kim et al. (Korea) [7]	☆	☆	☆	☆	☆		☆		☆
Lee et al.(Korea) [24]	☆	☆	☆	☆	☆		☆	☆	☆
Jiao et al .(China) [28]	☆	☆	☆	☆	☆		☆		☆
Nixon et al .(USA) [20]		☆	☆	☆		☆	☆		☆
Jin et al.(USA) [18]	☆	☆	☆		☆	☆	☆	☆	☆
Maia et al. (Brazil) [7]	☆	☆	☆	☆	☆	☆	☆	☆	☆
Moon et al.(Korea) [25]		☆	☆	☆	☆	☆	☆	☆	☆
Gerschpacher et al.(Austria) [16]		☆	☆	☆	☆	☆	☆	☆	☆

For cohort studies, 1 indicates exposed cohort truly representative; 2, nonexposed cohort drawn from the same community; 3, ascertainment of exposure; 4, outcome of interest not present at start; 5A, cohorts comparable on basis of age; 5B, cohorts comparable on other factor(s); 6, quality of outcome assessment; 7, follow-up long enough for outcomes to occur; and 8, complete accounting for cohorts.

Supplementary Table S2: Sensitivity analysis

Study omitted	Pooled ORs	95% CI
Boelaert et al.2006	2.00	1.63–2.47
Haymart et al.2007	2.01	1.63–2.48
Polyzos et al. 2008	2.02	1.62–2.52
Fiore et al. 2009	1.97	1.62–2.38
Fiore et al. 2010	2.07	1.68–2.54
Gul et al. 2010	1.93	1.58–2.36
Kim et al.2010	2.03	1.64–2.52
Ding et al.2011	1.99	1.62–2.45
Rio et al. 2011	2.07	1.67–2.57
Kim et al. 2012	2.03	1.63–2.53
Zafon et al. 2012	2.09	1.69–2.57
lee et al.2012	2.04	1.78–2.35
Zafon et al. 2014	2.02	1.64–2.49
Nixon et al.2014	1.93	1.58–2.36
Jiao et al.2015	1.99	1.62–2.45
Jin et al.2010	1.97	1.60–2.41
Moon et al.2012	2.01	1.63–2.48
Maia et al.2010	1.98	1.61–2.44
Gerschpacher et al. 2010	2.03	1.65–2.50
Combined	2.01	1.64–2.46
